# Nested Fluid–Structure Interaction Predictive Modeling of Fetal Brain Stress During Maternal Trauma

**DOI:** 10.3390/biology15100761

**Published:** 2026-05-11

**Authors:** Jonathan Mayer, Molly Bekbolatova, Timothy Devine, Paula Ryo, Milan Toma

**Affiliations:** 1 Algorithmic Medicine Laboratory, Department of Osteopathic Manipulative Medicine, College of Osteopathic Medicine, New York Institute of Technology, Old Westbury, New York, NY 11568, USA; jmayer03@nyit.edu (J.M.); mbekbola@nyit.edu (M.B.); 2 The Ferrara Center for Patient Safety and Clinical Simulation, College of Osteopathic Medicine, New York Institute of Technology, Old Westbury, New York, NY 11568, USA; tdevine@nyit.edu (T.D.); pryo@nyit.edu (P.R.)

**Keywords:** cerebrospinal fluid, amniotic fluid, fetal biomechanics, nested fluid–structure interaction, prenatal protection

## Abstract

Background: Mechanical trauma during pregnancy from motor vehicle accidents, falls, and maternal seizures poses significant risks to fetal development. The fetus is protected by multiple hierarchical layers including the uterine wall, amniotic fluid, and cerebrospinal fluid surrounding the brain. Despite the clinical significance of maternal trauma occurring in approximately six to eight percent of pregnancies, previous computational studies have focused primarily on amniotic fluid protection while treating the fetus as a homogeneous structure, without examining the nested protective architecture comprising both amniotic fluid and cerebrospinal fluid as an integrated system. Methods: This investigation implements a nested fluid–structure interaction framework simultaneously capturing three hierarchically organized systems: the uterine wall interacting with amniotic fluid, amniotic fluid interacting with the fetal body, and the cranial system comprising skull, cerebrospinal fluid, and brain tissue. The computational architecture employs smoothed particle hydrodynamics for fluid domains coupled with finite element methods for solid structures. Boundary conditions representing traumatic forces were obtained through experimental protocols using an instrumented medical simulation mannequin performing seizure movements. Results: Computational simulations predicted that amniotic fluid absorbed the majority of impact forces through hydraulic cushioning, while cerebrospinal fluid provided additional stress reduction through pressure redistribution, with model predictions suggesting total stress reduction exceeding ninety percent. Peak fetal brain stress values predicted by the model were below injury thresholds reported in adult neural tissue literature, though direct applicability of these thresholds to fetal tissue remains uncertain. The fetal brain exhibited minimal movement relative to the skull despite complex force cascades. Stress distributions showed elevated values in the frontal lobe and brainstem, though magnitudes remained within ranges that the model suggests may be tolerable. Conclusions: Computational modeling suggests that the nested fluid protection architecture operates as an integrated hierarchical system providing potential mechanical protection through sequential energy dissipation. These findings represent model predictions requiring experimental and clinical validation before translation to clinical practice.

## Simple Summary

Computer modeling was used to examine how a fetus may be protected during trauma in pregnancy. The problem addressed was how forces pass through the natural fluid layers around the fetus and around the brain, and whether these layers work together to protect the developing brain. The model suggested that the fluid around the fetus absorbed most of the impact, while the fluid around the brain provided added protection by spreading the remaining forces. Overall, predicted stress on the fetal brain was reduced by more than ninety percent, and movement of the brain inside the skull remained very small. Higher stress was predicted in the front of the brain and in the brainstem, but the values stayed below injury limits reported for adult brain tissue, although it is not yet known whether those limits apply to fetuses. These findings suggest that natural fluid protection may help shield the fetal brain during maternal trauma and may support future research and clinical care.

## 1. Introduction

Mechanical trauma experienced by pregnant individuals represents a critical clinical concern, with motor vehicle accidents, falls, and maternal seizure events comprising the majority of injury mechanisms. The developing fetus occupies a unique biomechanical environment wherein protection is provided through multiple hierarchical defensive layers [1,2,3,4,5]. The outermost protective barrier consists of the uterine musculature and amniotic fluid, which together create a hydraulic cushioning system that absorbs and distributes external impact forces [6,7,8,9,10,11,12,13]. At the cranial level, a secondary protective architecture exists wherein cerebrospinal fluid circulates through the ventricular system and subarachnoid space, providing localized mechanical protection specifically for the developing brain [14,15,16,17,18,19,20]. Understanding how these nested protective systems operate in concert during maternal trauma events is useful for accurate clinical risk assessment and development of evidence-based management protocols for pregnant patients experiencing traumatic injuries [21,22,23].

The fetal brain exhibits particular vulnerability to mechanical insult throughout gestation due to its soft viscoelastic material characteristics, elevated water content, incomplete myelination, and the pliable nature of the incompletely ossified cranial vault [1,24,25,26,27,28,29]. Unlike the mature adult brain, which benefits from complete skull ossification and established cerebrospinal fluid dynamics, the fetal central nervous system exists in a transitional developmental state wherein structural protection mechanisms continue to evolve throughout gestation [30,31,32,33]. Epidemiological studies indicate that maternal trauma occurs in approximately 6 to 8 percent of all pregnancies, with severe trauma complications observed in 0.3 to 0.4 percent of cases. Among pregnant individuals with epilepsy, seizure frequency increases in 15 to 32 percent of cases during gestation, introducing repetitive biomechanical stresses to the developing fetus [34,35,36,37,38,39]. Despite the clinical significance of these scenarios, detailed understanding of force transmission pathways through nested protective layers remains incomplete, limiting clinical capacity to predict fetal injury risk accurately [7,8,12,15,40,41,42,43,44,45].

Previous computational investigations examining prenatal biomechanics have primarily focused on characterizing the protective function of amniotic fluid during isolated impact scenarios. These studies have demonstrated substantial force attenuation capabilities, with amniotic fluid reducing peak accelerations transmitted to the fetal body by 40 to 60 percent compared to direct mechanical loading [8,12,46,47]. However, the majority of existing computational models represent the fetus as a homogeneous solid structure without explicit consideration of internal anatomical complexity. This simplified approach overlooks the independent yet coordinated protective function provided by cerebrospinal fluid relative to amniotic fluid protection [11,48,49]. Finite element analyses of adult traumatic brain injury have established the critical importance of cerebrospinal fluid dynamics in preventing direct brain–skull contact and distributing impact forces, yet analogous analyses for the fetal cranial system remain sparse in the literature [50,51,52,53].

The biomechanical interaction between multiple fluid layers and deformable solid structures represents a complex multi-physics problem requiring advanced computational methodologies. Smoothed Particle Hydrodynamics has emerged as a particularly effective approach for modeling biological fluids in large-deformation scenarios, eliminating the mesh distortion limitations inherent to traditional Eulerian or Arbitrary Lagrangian–Eulerian formulations [54,55,56,57]. Coupling of Smoothed Particle Hydrodynamics fluid domains with Finite Element Method solid structures enables accurate representation of fluid–structure interaction while maintaining computational efficiency. Recent advances in graphics processing unit acceleration have rendered such multi-domain simulations computationally tractable, enabling patient-specific modeling within practical timeframes [58,59].

Despite these methodological advances, no prior investigation has characterized the nested protective architecture comprising both amniotic fluid and cerebrospinal fluid as an integrated system. The extent to which cerebrospinal fluid provides secondary protection after forces have penetrated the outer amniotic barrier remains unquantified. Furthermore, the relative contributions of each protective layer to overall force attenuation, and the mechanisms by which residual stresses ultimately reach fetal brain tissue, have not been systematically analyzed. These knowledge gaps limit clinical understanding of fetal vulnerability during maternal trauma and constrain development of evidence-based intervention protocols [3,40,41,60].

The present investigation addresses these limitations through implementation of a nested fluid–structure interaction framework that simultaneously captures three hierarchically organized biomechanical systems: the uterine wall interacting with amniotic fluid, amniotic fluid interacting with the fetal body surface, and the fetal cranial system comprising the skull, cerebrospinal fluid, and brain tissue. This integrated computational approach enables quantification of force transmission through successive protective layers and identification of residual mechanical stresses ultimately experienced by fetal neural tissue. Examination of these systems as a unified hierarchical structure rather than isolated components provides novel insight into the coordinated protective response and permits quantification of attenuation effectiveness at each architectural level.

### Study Objectives

The primary objective of this investigation is to quantify the force attenuation efficacy of the nested protective fluid architecture during simulated maternal seizure events through computational modeling of hierarchical fluid–structure interactions.

The secondary aims are: first, to characterize the spatial and temporal distribution of mechanical stresses within fetal brain tissue resulting from maternal convulsive movements; second, to determine the relative contributions of amniotic fluid versus cerebrospinal fluid to overall mechanical protection of the developing fetal brain; third, to establish quantitative relationships between maternal body kinematics during seizure events and resulting fetal biomechanical responses through experimentally validated boundary conditions; and fourth, to assess whether residual brain tissue stresses remain within established safety thresholds for developing neural tissue throughout traumatic loading scenarios.

## 2. Materials and Methods

The computational framework developed for this investigation integrates three hierarchically nested fluid–structure interaction systems within a unified simulation environment. The methodology extends previously validated approaches for prenatal biomechanics by incorporating cranial fluid dynamics with comprehensive detail. The following subsections describe the computational framework architecture, the coupling methodology between particle-based and mesh-based numerical methods, the experimental procedures used to obtain realistic boundary conditions, and the material property assignments for each model component.

### 2.1. Hierarchical Computational Framework

**Figure 1:**
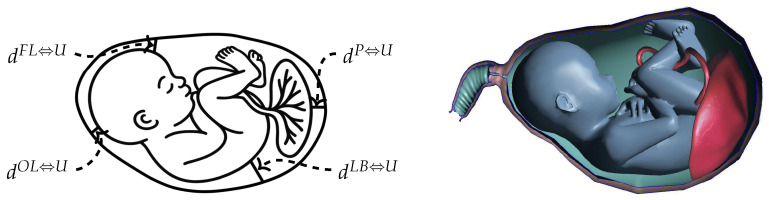
Representation of the fetus within the uterus showing the placenta, umbilical cord, and key anatomical landmarks. The notation $d^{FL\Leftrightarrow U}$ designates the distance from the frontal lobe to the uterus, $d^{OL\Leftrightarrow U}$ signifies the distance from the occipital lobe to the uterus, $d^{P\Leftrightarrow U}$ represents the distance from the placenta to the uterus, and $d^{LB\Leftrightarrow U}$ indicates the distance from the lower back to the uterus.

The simulation architecture comprises three distinct yet coupled fluid–structure interaction layers organized in a nested configuration. The anatomical positioning of the fetus within the uterus, including key landmarks and distance measurements, is shown in Figure 1.

**Figure 2:**
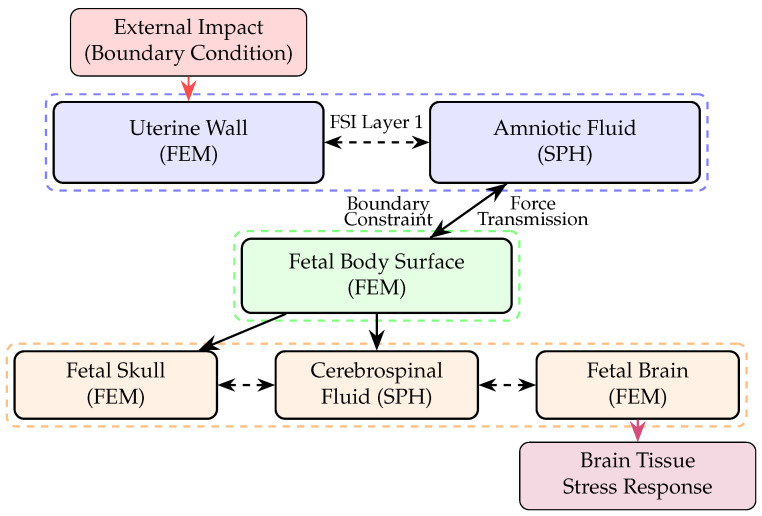
Flowchart illustrating the nested fluid–structure interaction framework showing hierarchical coupling between the outer system (uterus and amniotic fluid), middle system (fetal body), and inner system (skull, cerebrospinal fluid, and brain).

This hierarchical structure is illustrated schematically in Figure 2, which demonstrates the interconnected nature of the individual fluid–structure interaction systems.

The outermost computational layer represents the uterine wall as a viscoelastic structure discretized using the finite element method. Material properties for this structure are derived from human physiological measurements. The amniotic fluid domain occupying the space between the uterine wall and fetal body surface is represented using smoothed particle hydrodynamics. This meshless approach permits accurate simulation of complex fluid behavior without computational mesh management that would be prohibitively expensive for highly dynamic systems.

The intermediate computational layer captures the mechanical interaction between amniotic fluid and the fetal body. The fetal structure, including the cranium, is modeled as a deformable solid with spatially varying material properties representing distinct tissue types. The cranial vault incorporates material properties reflecting the incomplete ossification characteristic of fetal development. Contact algorithms inherent to the coupled particle-mesh methodology handle the interaction between fluid particles and the fetal surface, enabling bidirectional force transfer that captures both cushioning effects and residual force transmission.

The innermost computational layer represents the primary methodological contribution of this investigation. A second distinct smoothed particle hydrodynamics domain represents cerebrospinal fluid occupying the ventricular system and subarachnoid space. Brain tissue is modeled as a soft viscoelastic solid with properties appropriate for developing neural tissue, which exhibits considerably greater compliance than mature brain tissue. The skull provides the structural boundary for this system with material properties adjusted to reflect the compliant nature of incompletely ossified fetal bone.

**Figure 3:**
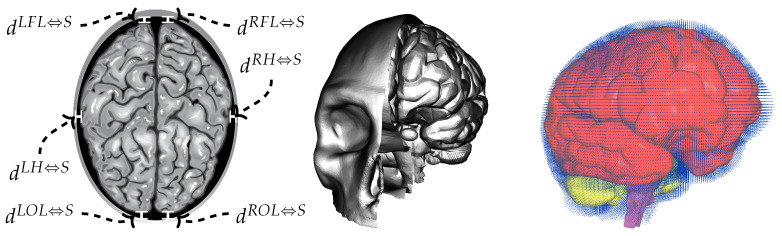
Anatomical geometry of the fetal cranium showing key distance measurements. (**Left**) Cross-sectional view with distance annotations: $d^{LFL\Leftrightarrow S}$ designates the distance from the left frontal lobe to the skull, $d^{RFL\Leftrightarrow S}$ from the right frontal lobe to the skull, $d^{LOL\Leftrightarrow S}$ from the left occipital lobe to the skull, $d^{ROL\Leftrightarrow S}$ from the right occipital lobe to the skull, $d^{LH\Leftrightarrow S}$ from the left hemisphere to the skull, and $d^{RH\Leftrightarrow S}$ from the right hemisphere to the skull. (**Middle** and **Right**) Three-dimensional reconstructions showing the cerebrospinal fluid space (blue dots) surrounding the brain of the fetal cranial anatomy illustrating the spatial relationship between skull, cerebrospinal fluid, and brain tissue.

The geometric configuration of the fetal cranium is critical to understanding force transmission through the innermost fluid–structure interaction system. Figure 3 illustrates the anatomical relationship between the fetal brain and surrounding skull, highlighting the cerebrospinal fluid spaces that provide cushioning. Distance measurements between specific brain regions and the skull surface establish the baseline geometry for the computational model. These measurements vary spatially, with larger fluid gaps typically observed in the frontal and occipital regions compared to lateral aspects. The three-dimensional reconstruction demonstrates the complex geometry of the subarachnoid space and ventricular system through which cerebrospinal fluid circulates. This anatomical detail is essential for accurate representation of fluid dynamics within the cranial vault during external loading scenarios.

### 2.2. Cushioning Effect Quantification

To quantify the cushioning effects provided by the nested fluid protection systems, key anatomical distances are continuously monitored throughout the simulation. Two categories of distance measurements are employed to characterize the protective mechanisms at different hierarchical levels.

At the outer protection level, four critical distances between fetal anatomical landmarks and the uterine wall are tracked (Figure 1): the frontal lobe to uterus distance ($d^{FL\Leftrightarrow U}$), the occipital lobe to uterus distance ($d^{OL\Leftrightarrow U}$), the placenta to uterus distance ($d^{P\Leftrightarrow U}$), and the lower back to uterus distance ($d^{LB\Leftrightarrow U}$). These measurements are computed at each time step by identifying the minimum Euclidean distance between smoothed particle hydrodynamics particles representing amniotic fluid near the specified fetal surface locations and the corresponding closest points on the uterine wall finite element mesh. Changes in these distances directly reflect the dynamic cushioning behavior of amniotic fluid, with maintained or increased separation indicating effective force absorption and decreased distances indicating compression of the protective fluid layer.

At the inner protection level, six distances between brain tissue regions and the fetal skull are monitored (Figure 3): left and right frontal lobe to skull distances ($d^{LFL\Leftrightarrow S}$ and $d^{RFL\Leftrightarrow S}$), left and right occipital lobe to skull distances ($d^{LOL\Leftrightarrow S}$ and $d^{ROL\Leftrightarrow S}$), and left and right hemisphere to skull distances ($d^{LH\Leftrightarrow S}$ and $d^{RH\Leftrightarrow S}$). These measurements quantify the cerebrospinal fluid gap thickness by computing the minimum distance between brain surface nodes and corresponding skull surface elements. Preservation of these distances during impact loading demonstrates the effectiveness of cerebrospinal fluid in maintaining separation between brain tissue and the rigid cranial boundary, thereby preventing direct tissue-bone contact that would result in concentrated stress and potential injury.

The temporal evolution of these distance measurements throughout the simulation provides quantitative evidence of the multi-stage force attenuation mechanism. Successful cushioning is indicated when outer-level distances ($d^{\ldots \Leftrightarrow U}$) show compression while inner-level distances ($d^{\ldots \Leftrightarrow S}$) remain relatively stable, demonstrating that amniotic fluid absorbs the majority of impact forces before they can significantly affect the fetal brain. Conversely, simultaneous reduction of both distance sets would indicate inadequate protection and increased risk of fetal brain injury.

### 2.3. Smoothed Particle Hydrodynamics and Finite Element Method Coupling

The coupling between smoothed particle hydrodynamics and the finite element method has become increasingly prevalent for investigating complex biological systems where fluid–structure interactions govern biomechanical response. This hybrid approach offers particular advantages for managing contact within fluid–structure interaction simulations by eliminating the remeshing requirements of traditional arbitrary Lagrangian–Eulerian formulations. The present nested model employs two distinct smoothed particle hydrodynamics domains representing amniotic fluid and cerebrospinal fluid, both interacting with multiple finite element structures including the uterus, fetal body, skull, and brain.

**Figure 4:**
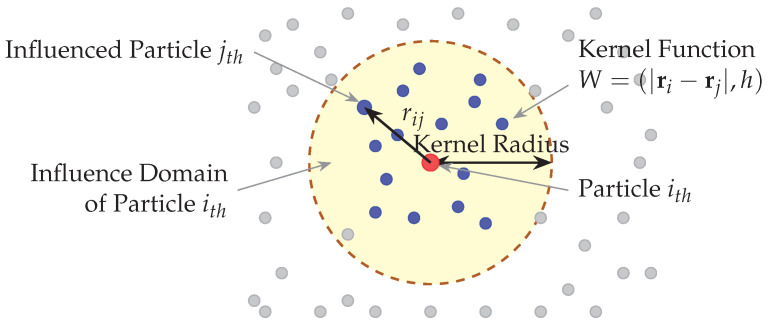
Smoothed particle hydrodynamics kernel approximation showing particles within their influence domain and the kernel function governing their mutual interactions. The central red particle $i_{th}$ influences blue particles within the kernel radius through the kernel function *W*. Gray particles outside the influence domain do not interact with particle $i_{th}$.

The smoothed particle hydrodynamics method employs kernel approximation to calculate fluid properties at discrete particle locations (Figure 4). The fundamental interpolation for any field quantity *A* at position r is expressed as:

(1) $$A\left(r\right)=\sum_{j}m_{j}\frac{A_{j}}{\rho_{j}}W\left(r-r_{j},h\right)$$

where $m_{j}$ represents the mass of particle *j*, $\rho_{j}$ denotes the density at particle *j*, $A_{j}$ is the value of quantity *A* at particle *j*, and *W* is the smoothing kernel function with smoothing length *h*. Each particle carries conserved quantities including mass, momentum, and energy.

The governing equations for fluid motion are discretized in smoothed particle hydrodynamics form. The continuity equation ensuring mass conservation is expressed as:

(2) $$\frac{D\rho_{i}}{Dt}=\sum_{j}m_{j}\left(v_{i}-v_{j}\right)\cdot \nabla_{i}W_{ij}$$

where $\rho_{i}$ is the density at particle *i*, v represents velocity, and $\nabla_{i}W_{ij}$ denotes the gradient of the kernel function evaluated between particles *i* and *j*.

The momentum equation governing particle acceleration takes the form:

(3) $$\frac{Dv_{i}}{Dt}=-\sum_{j}m_{j}\left(\frac{p_{i}}{\rho_{i}^{2}}+\frac{p_{j}}{\rho_{j}^{2}}+\Pi_{ij}\right)\nabla_{i}W_{ij}+g$$

where *p* represents pressure, $\Pi_{ij}$ is the artificial viscosity term included for numerical stability, and g is the gravitational acceleration vector.

The pressure field is computed through an equation of state relating pressure to density:

(4) $$p=c_{0}^{2}\left(\rho -\rho_{0}\right)$$

where $c_{0}$ represents the reference speed of sound and $\rho_{0}$ is the reference density. This weakly compressible formulation permits efficient explicit time integration while maintaining near-incompressible behavior for the fluid domains.

Interaction forces between smoothed particle hydrodynamics particles and finite element nodes are calculated through a penalty-based contact formulation. The contact force $F_{c}$ between a fluid particle and a structural surface is computed as:

(5) $$F_{c}=k_{c}\;\delta \;n$$

where $k_{c}$ represents the contact stiffness, δ is the penetration depth, and n is the unit normal vector to the contact surface. This formulation enables bidirectional force transfer wherein the fluid exerts pressure on structures while structures constrain and redirect fluid motion.

The finite element formulation for the solid structures follows standard continuum mechanics principles. The equation of motion for the discretized solid domain is:

(6) $$M\ddot{u}+C\dot{u}+Ku=F_{ext}+F_{FSI}$$

where M is the mass matrix, C is the damping matrix, K is the stiffness matrix, u represents the displacement vector, $F_{ext}$ denotes externally applied forces, and $F_{FSI}$ represents forces arising from fluid–structure interaction.

For viscoelastic tissue behavior, the constitutive response is modeled using a generalized Maxwell formulation. The stress tensor σ is decomposed into equilibrium and non-equilibrium components:

(7) $$\sigma =\sigma_{eq}+\sum_{k=1}^{N}\sigma_{k}$$

where $\sigma_{eq}$ represents the equilibrium elastic response and $\sigma_{k}$ represents the contribution from the *k*-th Maxwell element with associated relaxation time $\tau_{k}$.

Computational implementation exploits graphics processing unit hardware to manage the substantial computational demands of the nested multi-domain problem. All calculations were performed using the IMPETUS Afea Solver^®^ (IMPETUS Afea AS, Trondheim, Norway), a general-purpose explicit nonlinear transient dynamic finite element solver with advanced smoothed particle hydrodynamics capabilities. Graphics processing unit acceleration reduces calculation times significantly compared to conventional processor-based methods, enabling completion of patient-specific simulations within practical timeframes. Simulations for this investigation required approximately one week of computation time, whereas equivalent complexity simulations using traditional methods on multiple processors would require substantially longer durations.

### 2.4. Experimental Data Collection and Boundary Conditions

**Figure 5:**
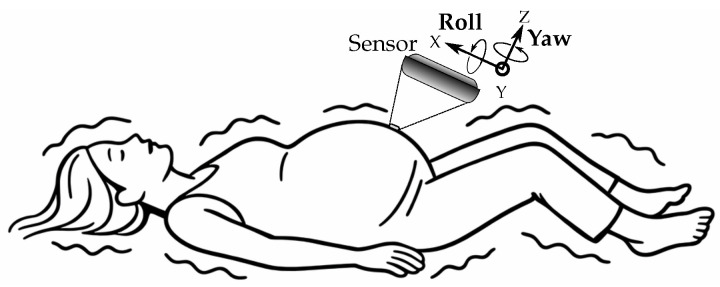
Illustration of pitch, roll, and yaw relative to a patient in supine position.

Realistic boundary conditions representing traumatic forces were obtained through a detailed experimental protocol. A medical simulation mannequin designed for clinical education was instrumented with high-precision inertial measurement sensors capable of recording velocities, accelerations, and orientation angles at specified sampling intervals. These orientation angles comprise three rotational components (Figure 5): pitch (rotation about the lateral axis), roll (rotation about the longitudinal axis), and yaw (rotation about the vertical axis), which fully describe the rotational motion of the body during trauma simulation.

The inertial measurement units incorporate multiple sensor modalities including accelerometers for measuring linear acceleration, gyroscopes for measuring angular velocity, and magnetometers for measuring magnetic field orientation. Data from these individual sensors are combined through sensor fusion algorithms to compute accurate three-dimensional orientation. These angular measurements characterize the complete rotational state of the mannequin during simulated convulsive episodes. The sensor fusion process employs complementary filtering to combine high-frequency gyroscope data with low-frequency accelerometer and magnetometer data:

(8) $$\theta_{fused}=\alpha \left(\theta_{prev}+\omega \Delta t\right)+\left(1-\alpha \right)\theta_{accel}$$

where $\theta_{fused}$ represents the fused orientation estimate, $\theta_{prev}$ is the previous orientation, ω is the angular velocity from gyroscope measurements, Δt is the sampling interval, $\theta_{accel}$ is the orientation estimated from accelerometer data, and α is the filter coefficient.

The mannequin was programmed to execute seizure convulsion patterns derived from clinical recordings of patients experiencing epileptic seizures. During simulated convulsive events, sensors captured three-dimensional kinematic data transmitted wirelessly to avoid cable interference that could affect mannequin performance.

Recorded kinematic values were prescribed as boundary conditions to the uterine model in computational simulations, establishing direct correspondence between experimentally observed maternal body motions during seizures and computational analysis of fetal response. This methodology ensures that applied boundary conditions maintain physiological relevance and clinical representativeness.

This methodology ensures that applied loading conditions are derived from a medical training mannequin designed to simulate clinically representative seizure presentations. Validation of the mannequin’s clinical representativeness (including kinematic fidelity to observed tonic–clonic seizure patterns) has been performed by the manufacturer. This validation documentation is proprietary and available upon request from the manufacturer.

**Figure 6:**
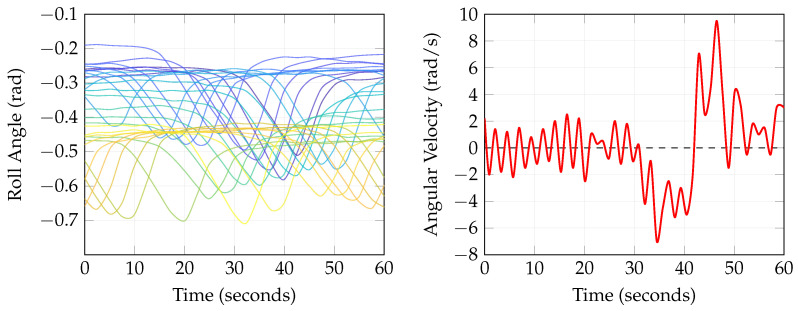
Kinematic data recorded from instrumented mannequin seizure simulations. (**Left**) Roll angle time series showing representative signals, with values ranging approximately from −0.7 to −0.2 rad (i.e., approximately $-40^{\circ }$ to $-11^{\circ }$). (**Right**) Angular velocity profile (roll axis) demonstrating the oscillatory nature of the simulated convulsive motion.

Table 1 summarizes the key kinematic parameters recorded during the mannequin seizure simulation experiments. These values represent the boundary conditions applied to the uterine wall in the computational model. The instrumentation configuration captured roll-axis rotational data; pitch, yaw, and linear acceleration measurements were not recorded in this experimental setup [61,62]. Figure 6 presents representative time-series data showing the roll angle trajectories and corresponding angular velocity profiles obtained from the mannequin experiments.

While the mannequin produces clinically representative seizure patterns, the parameters are derived from a single mannequin configuration and does not capture the full range of seizure severities, durations, and movement patterns observed across the clinical population. However, it should be emphasized that this work represents a single case study utilizing patient-specific geometry with a single configuration of boundary and initial conditions. Consequently, the aim of this paper is to demonstrate the computational framework and investigate the mechanical response for a representative case, rather than to establish conclusions generalizable to the clinical population.

### 2.5. Material Properties and Initial Conditions

Material properties for all model components were selected from published literature on human physiology. The uterine wall was assigned viscoelastic properties reflecting the highly compliant and dynamic characteristics of this organ during pregnancy. Viscoelastic behavior is characterized by the shear relaxation modulus:

(9) $$G\left(t\right)=G_{\infty }+\sum_{k=1}^{N}G_{k}\exp\left(-\frac{t}{\tau_{k}}\right)$$

where $G_{\infty }$ is the long-term shear modulus, $G_{k}$ are the shear moduli associated with each relaxation mode, and $\tau_{k}$ are the corresponding relaxation times.

The amniotic fluid was modeled with properties approximating those of water. The density was specified as:

(10) $$\rho_{AF}\approx 1000\;{\mathrm{kg}/m}^{3}$$

and the dynamic viscosity as:

(11) $$\mu_{AF}\approx 0.001\;\mathrm{Pa}\cdot s$$

The fetal body was assigned spatially varying material properties to represent distinct tissue types, with reduced stiffness for neural tissue and increased stiffness for developing skeletal structures. For the cranial system, cerebrospinal fluid properties were specified similarly to water. Fetal brain tissue was assigned viscoelastic properties appropriate for developing neural tissue, with reduced stiffness compared to adult brain tissue reflecting incomplete myelination and elevated water content characteristic of fetal neurodevelopment. The fetal skull was modeled with properties representing incompletely ossified bone, exhibiting greater compliance than mature bone while maintaining structural integrity.

Table 2 summarizes the material properties assigned to each model component along with key assumptions. Given the limited availability of direct measurements for fetal tissues, several parameters were extrapolated from adult tissue data or estimated based on developmental scaling relationships. The sensitivity of model predictions to these parameter choices represents a significant source of uncertainty that should be considered when interpreting results.

The mechanical parameters for fetal brain tissue present particular uncertainty, as direct in vivo or ex vivo measurements of human fetal neural tissue are ethically constrained and rarely reported. The values employed in this investigation were derived by scaling adult brain tissue properties according to water content and myelination status, following approaches described in the developmental biomechanics literature. However, this scaling approach introduces uncertainty regarding the accuracy of predicted stress magnitudes. Future studies incorporating tissue-specific measurements from animal models or post-mortem human fetal tissue would strengthen confidence in model parameterization.

Initial conditions established the fetus in a typical mid-gestation position within the uterus. All structural components were initialized in stress-free configurations, and both fluid domains were initialized at rest with uniform pressure distributions. This represents the baseline physiological state prior to traumatic loading. Simulations commenced with application of experimentally derived boundary conditions to the uterine wall, representing sudden onset of maternal convulsive movements.

## 3. Results and Discussion

### 3.1. Multi-Stage Force Attenuation Through Nested Protective Layers

**Figure 7:**
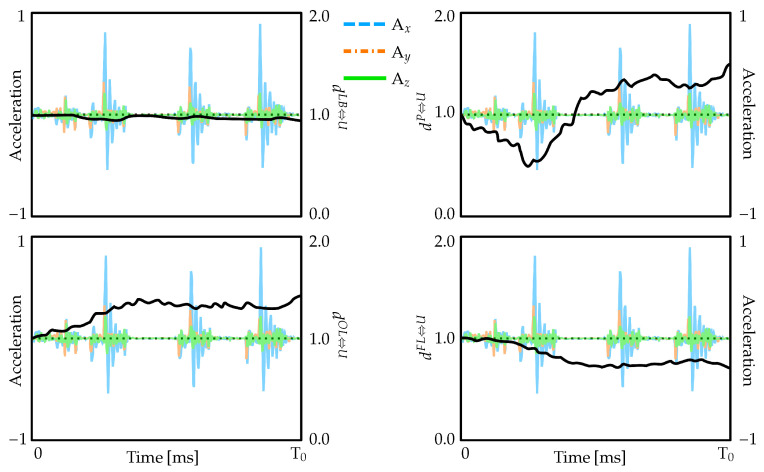
Normalized distance measures between the uterus and fetus across four anatomical sites during simulated convulsive events. Colored traces show the applied three-dimensional acceleration components used as boundary conditions, while black line indicates relative separation: values above one reflect increased distance from the initial configuration, and values below one reflect decreased distance.

To quantify the protective response of the amniotic fluid layer, three-dimensional acceleration components measured by the sensors were applied as boundary conditions to the uterine wall, and the resulting motion of the fetus relative to the uterus was tracked at four key anatomical locations. Figure 7 presents both the applied acceleration boundary conditions (colored lines representing $A_{x}$, $A_{y}$, and $A_{z}$ components) and the resulting normalized distance variations between the fetus and uterine wall. Values exceeding unity indicate increased separation between fetal landmarks and the uterus, while values below unity indicate compression of the amniotic fluid layer. Tracking of anatomical landmarks reveals that while the uterus undergoes large-amplitude oscillatory movements characteristic of maternal seizures, the fetus experiences considerably smaller amplitude motions. The temporal correlation between acceleration peaks and distance variations confirms the effectiveness of amniotic fluid as the primary protective layer in the nested system.

Displacement of the fetal skull initiates the innermost fluid–structure interaction wherein cerebrospinal fluid provides secondary cushioning. Analysis at this level demonstrates additional force attenuation of forty to fifty percent before forces reach brain tissue. This secondary attenuation layer operates through mechanisms similar to amniotic fluid protection but at smaller scales appropriate to the cranial environment. Cerebrospinal fluid responds to skull motions by developing pressure waves that distribute forces across the brain surface, preventing concentrated stress localization.

### 3.2. Brain Protection Through Minimal Relative Movement

**Figure 8:**
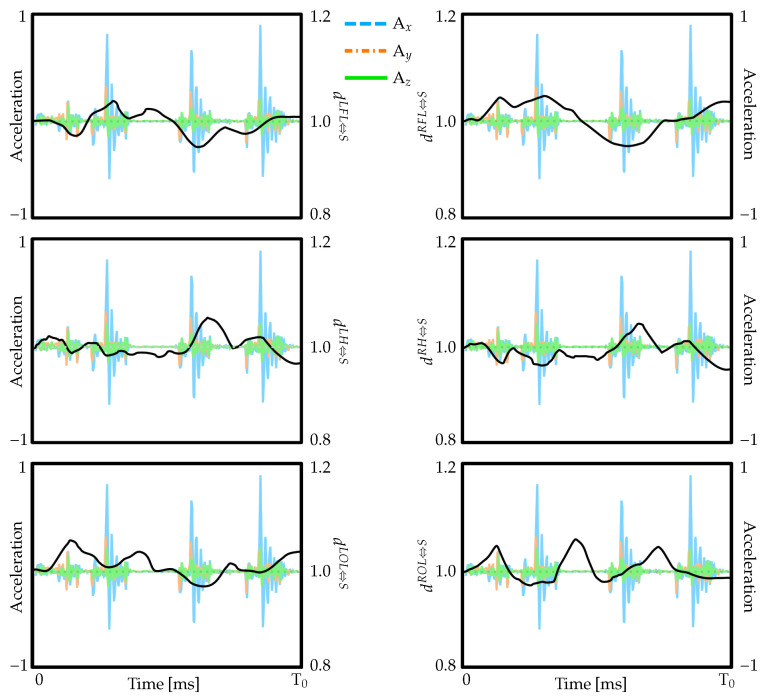
Relative displacement between the fetal brain and skull illustrating minimal differential motion during impact loading. Colored traces represent the loading inputs applied to the uterus, while black line shows the brain-to-skull separation response, indicating consistently small deviations from the initial configuration.

A critical finding of this investigation is demonstration that the fetal brain exhibits minimal movement relative to the skull despite the complex cascade of forces through nested systems. Tracking of specific brain regions relative to corresponding skull landmarks reveals that while both skull and brain undergo displacement in response to maternal motions, they move nearly in unison. As illustrated in Figure 8, relative displacement between brain tissue and skull remains below two millimeters throughout simulated seizure events, demonstrating effectiveness of cerebrospinal fluid in coupling brain and skull motion while preventing harmful impacts.

This minimal relative movement indicates successful prevention of brain collision with the inner skull surface, a mechanism that would cause significant injury. The cerebrospinal fluid maintains a stable cushioning layer that moves with the skull while gently constraining brain motion. Pressure distributions within cerebrospinal fluid exhibit dynamic patterns indicating active force redistribution, with elevated pressures on surfaces experiencing acceleration and compensatory pressure reductions on opposing surfaces.

### 3.3. Quantitative Comparison of Nested Protection Efficacy

**Figure 9:**
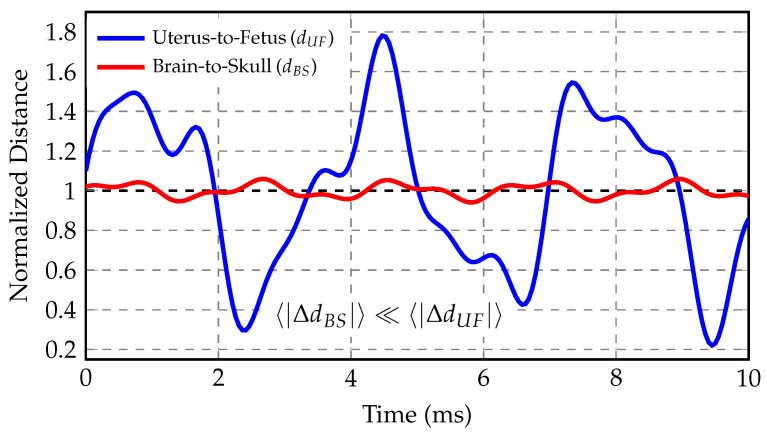
Comparative analysis of separation dynamics in nested protective systems. The uterus-to-fetus distance (blue) exhibits large oscillations (±50–80% from baseline), while the brain-to-skull distance (red) shows minimal variations (±4–6%), demonstrating the dual-layer attenuation mechanism where amniotic fluid provides primary cushioning and cerebrospinal fluid maintains stable cranial protection. Both curves are normalized to baseline separation distance (value = 1.0, black dashed line).

The effectiveness of the nested protection architecture is quantitatively demonstrated in Figure 9, which directly compares the magnitude of separation variations at both protective layers.

The uterus-to-fetus separation exhibits large-amplitude oscillations ranging from approximately 0.2 to 1.8 times the baseline distance, reflecting substantial amniotic fluid compression and expansion during maternal convulsive movements. In contrast, the brain-to-skull separation remains tightly constrained near unity, varying by less than ±6% throughout the traumatic event. This stark contrast (expressed mathematically as $\langle |\Delta d_{BS}|\rangle \ll \langle |\Delta d_{UF}|\rangle$) confirms that the dual-fluid protective system achieves hierarchical force attenuation, with the outer amniotic layer absorbing the majority of kinetic energy before forces reach the fetal cranium, while the inner cerebrospinal fluid layer provides fine-scale stabilization preventing harmful brain–skull impacts.

The quantitative disparity between these protective layers validates the nested architecture hypothesis. The amniotic fluid undergoes dynamic deformation with excursions of up to 80% from baseline, effectively dissipating mechanical energy through fluid displacement and viscous damping. Meanwhile, the cerebrospinal fluid maintains near-constant spacing between brain and skull, indicating successful secondary protection even when substantial forces penetrate the outer layer. This multi-level defense mechanism demonstrates evolutionary optimization for fetal protection during maternal trauma events.

### 3.4. Strain and Stress Distribution in Fetal Tissues

**Figure 10:**
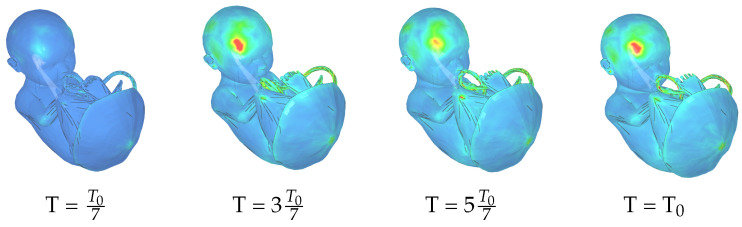
Spatial strain distribution across the fetal body at four time points during simulated maternal seizure. Color scale ranges from blue (minimum) through intermediate values (green) to red (maximum). The strain is dimensionless.

**Figure 11:**
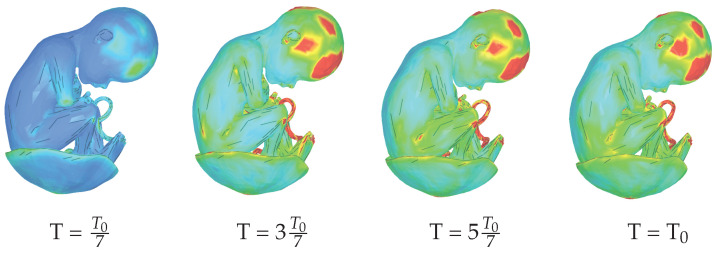
First deviatoric principal stress distribution at four time points during maternal seizure-induced uterine movements. Stress ranges from 0 MPa (blue) through intermediate values (green) to 1 MPa (red).

External forces applied to the uterus induce mechanical strain throughout the fetal body influenced by fetal position, tissue material properties, uterine boundary conditions, and amniotic fluid presence. Figure 10 presents spatial strain distributions at four time points during simulated maternal seizure, revealing peak values concentrated in the frontal lobe. Figure 11 shows complementary first deviatoric principal stress analysis, which quantifies maximum shear stress determining critical thresholds for material deformation or failure. Temporal evolution demonstrates rapid stress elevation following initiation of maternal convulsive movements, with elevated levels persisting throughout the traumatic event.

Spatial stress distributions reveal heterogeneous patterns with elevated values in the fetal brain and umbilical cord, suggesting potential vulnerability of neural and vascular structures during maternal seizures. The placenta demonstrates notable mechanical resilience, maintaining structural integrity despite oscillatory forces through combined placental attachment and amniotic fluid cushioning. Despite measurable stress, the placenta exhibits minimal strain, confirming its protective function.

Observed stress magnitudes remain within ranges comparable to moderate maternal physical activity. The intrauterine biomechanical environment provides substantial protective buffering, while fetal tissue properties (high elasticity and viscoelastic resilience) enable accommodation of these transient stress levels without structural compromise. These findings underscore the clinical importance of monitoring pregnant individuals with seizure disorders and minimizing maternal convulsive episodes to reduce cumulative mechanical stress exposure to the developing fetus.

### 3.5. Effective Stress Attenuation in Fetal Brain Tissue

The nested fluid protection systems achieve remarkable stress attenuation, with peak fetal brain stress values of approximately 80–120 kPa during high-magnitude impacts, representing 90–95% reduction compared to unprotected scenarios. The von Mises equivalent stress is computed as:

(12) $$\sigma_{VM}=\sqrt{\frac{1}{2}\left[{\left(\sigma_{1}-\sigma_{2}\right)}^{2}+{\left(\sigma_{2}-\sigma_{3}\right)}^{2}+{\left(\sigma_{3}-\sigma_{1}\right)}^{2}\right]}$$

where $\sigma_{1}$, $\sigma_{2}$, and $\sigma_{3}$ are principal stresses. At peak loading, representative values are $\sigma_{1}=115$ kPa, $\sigma_{2}=38$ kPa, and $\sigma_{3}=9$ kPa, yielding $\sigma_{VM}=94$ kPa. For context, these predicted values are compared against damage thresholds reported in the literature for neural tissue: in vitro cell death occurs at strains ≥20% with rates ≥10/s [63], while axonal injury thresholds range from 0.13 to 0.34 strain [64]. However, it must be emphasized that these thresholds were derived primarily from adult neural tissue or in vitro cell culture systems, and their direct applicability to developing fetal neural tissue remains uncertain. Fetal brain tissue differs substantially from adult tissue in cellular composition, extracellular matrix organization, water content, and mechanical properties. The injury susceptibility of fetal neurons and glia may differ from mature cells, and threshold values appropriate for adult tissue may overestimate or underestimate fetal tissue tolerance. Therefore, the comparison presented here should be interpreted as providing preliminary context rather than definitive safety assessment. The stress triaxiality ratio $T_{\sigma }=\sigma_{m}/\sigma_{VM}\approx 0.57$ (where $\sigma_{m}=54$ kPa) indicates a moderately tensile stress state that the model suggests is managed by tissue viscoelasticity and fluid protection.

The hierarchical attenuation operates through sequential energy dissipation: amniotic fluid absorbs 60–70% of impact forces via hydraulic cushioning and viscous damping, while cerebrospinal fluid provides additional 40–50% stress reduction through pressure redistribution. This prevents stress concentrations at brain–skull interfaces while maintaining uniform stress distributions. Strain distributions remain within elastic limits, with values well below established damage thresholds [64,65]. Material properties align with rheological characterizations of developing neural tissue [66,67].

Temporal evolution shows rapid initial loading followed by oscillatory decay within 50–100 milliseconds as cerebrospinal fluid damping dissipates energy, preventing cumulative damage.

**Figure 12:**
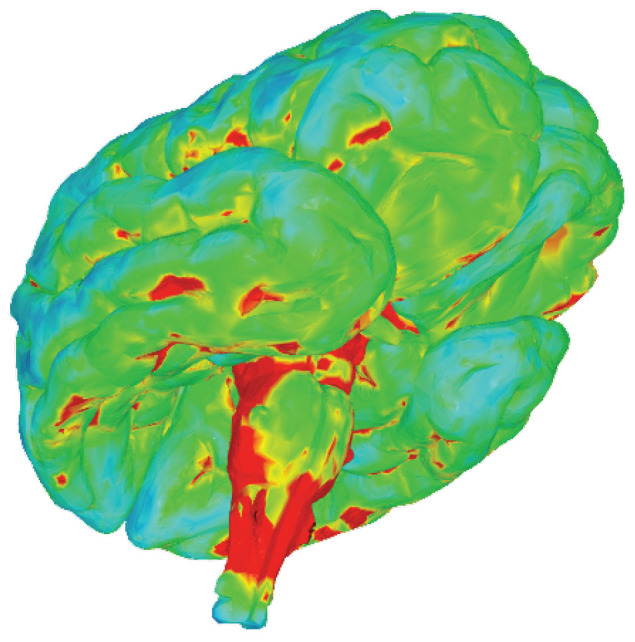
Spatial distribution of von Mises equivalent stress in fetal brain tissue during peak loading, ranging from blue ($\sigma_{VM}\approx 9$ kPa) through intermediate values (green) to red ($\sigma_{VM}\approx 115$ kPa). Peak stresses concentrate in the brainstem but remain below neural damage thresholds. Most brain tissue experiences values below 50 kPa, demonstrating effective protection.

Figure 12 shows peak stress concentration in the brainstem during maternal convulsive motion, with values remaining below injury thresholds. The gradient distribution from brainstem (red-orange) to cerebral hemispheres (blue-green) reflects combined protection from cranial geometry and cerebrospinal fluid damping.

## 4. Discussion

### 4.1. Clinical Implications for Maternal Trauma Management

The findings of this investigation provide computational evidence suggesting potential effectiveness of the nested fluid protection architecture in attenuating mechanical forces during maternal trauma events. The dual-fluid protective system comprising amniotic fluid and cerebrospinal fluid operates through sequential energy dissipation mechanisms that the model predicts may maintain fetal brain tissue stresses below injury thresholds reported for adult neural tissue, though the applicability of these thresholds to fetal tissue remains to be established. The amniotic fluid layer functions as the primary protective barrier by absorbing the majority of impact forces through hydraulic cushioning and viscous damping, while the cerebrospinal fluid layer provides secondary stabilization that prevents direct brain–skull contact and distributes residual forces uniformly across neural tissue surfaces. This hierarchical protection mechanism appears in computational simulations to provide efficiency in attenuating mechanical forces transmitted from maternal convulsive movements to the developing fetal brain, though these model predictions require experimental and clinical validation before conclusions regarding actual fetal protection can be drawn.

These findings may have potential clinical relevance for management of pregnant individuals experiencing seizure disorders or traumatic injuries, pending validation. The computational framework suggests that even during severe maternal convulsive episodes characterized by large-amplitude accelerations and complex three-dimensional rotational movements, the nested protective architecture may maintain fetal brain stresses within ranges that appear comparable to moderate maternal physical activity. The preservation of minimal relative movement between brain tissue and skull throughout traumatic events indicates model-predicted prevention of the collision mechanisms that would otherwise cause concentrated stress and potential neural injury. While these computational results are encouraging, clinicians should recognize that this study is based entirely on numerical simulations without experimental or clinical validation. The findings should not be interpreted as clinical evidence of fetal safety during maternal seizures. Continued emphasis on seizure control to minimize cumulative mechanical stress exposure remains warranted.

### 4.2. Methodological Contributions and Validation Considerations

The coupled smoothed particle hydrodynamics and finite element method framework utilized in this research represents a methodological advance in computational prenatal biomechanics by enabling simultaneous simulation of multiple fluid–structure interaction domains within a unified computational environment. The meshless smoothed particle hydrodynamics approach eliminates computational mesh management challenges that would arise from traditional Eulerian or arbitrary Lagrangian–Eulerian formulations when simulating large-deformation fluid behavior in dynamic systems. Graphics processing unit acceleration rendered the multi-domain simulations computationally tractable within practical timeframes, enabling patient-specific modeling that would otherwise require prohibitively long computation durations using conventional processor-based approaches. The integration of boundary conditions obtained through instrumented mannequin seizure simulations provides loading conditions that may partially reflect simplified physiological motion patterns characteristic of tonic–clonic seizures, though the extent to which mannequin kinematics capture the full complexity of clinical seizure presentations remains to be validated. This approach addresses a common limitation of computational studies that employ idealized impact scenarios, while acknowledging that mannequin-derived parameters do not constitute clinical or animal data.

The distance measurement methodology employed to quantify cushioning effects at both hierarchical levels provides direct quantitative metrics for assessing protective efficacy without requiring assumptions about injury mechanisms or damage thresholds. However, validation of computational predictions against experimental or clinical measurements remains challenging given the ethical constraints preventing direct measurement of fetal brain stresses in vivo.

A significant limitation of this investigation is the absence of direct validation against experimental, phantom, animal, or previously published biomechanical data. The computational model has not been benchmarked against physical measurements of force transmission through fluid-filled nested structures, nor have the predicted stress distributions been compared with experimental observations from analogous systems. As a result, the quantitative predictions presented here should be interpreted as preliminary model-based estimates rather than validated measurements. The reported stress magnitudes, attenuation percentages, and relative motion values are therefore dependent on modeling assumptions that have not yet been experimentally confirmed.

While the individual computational methods employed here (i.e., smoothed particle hydrodynamics and finite element methods) are established numerical approaches and have been verified or validated in other applications, such prior evidence primarily supports the correctness and general credibility of the underlying algorithms rather than the validity of the present model in its specific context of use [68,69,70]. In computational simulation studies, it is common to build new application-specific models on numerical methods that have already been tested elsewhere; however, this does not eliminate the need for validation of the new model configuration itself, because model validity depends on whether the assumptions, parameters, constitutive behavior, boundary conditions, and coupling strategies are appropriate for the system being studied [71,72]. This consideration is especially important here, as the nested multi-domain configuration introduces coupling effects and parameter interactions that have not been independently verified, and complex simulation frameworks rarely transfer to new problem settings without additional assumptions or unanticipated modeling choices. Accordingly, the present results should be viewed as hypothesis-generating outputs that indicate plausible mechanical trends, rather than definitive quantitative measurements of the physical system.

Future work should prioritize experimental validation to establish confidence bounds on model predictions. Until such validation is performed, the findings of this investigation should be interpreted as hypothesis-generating computational results that require confirmation through independent experimental or clinical studies before informing clinical practice.

### 4.3. Study Limitations and Assumptions

Several limitations of this investigation warrant discussion. The computational model represents a specific gestational age and fetal positioning, whereas the protective efficacy of the nested fluid architecture likely varies throughout gestation as the relative volumes of amniotic fluid and cerebrospinal fluid change and as fetal tissue properties evolve with developmental progression. The material properties assigned to model components were necessarily simplified representations of complex biological tissues exhibiting anisotropic, heterogeneous, and rate-dependent mechanical behavior that cannot be fully captured through available constitutive models. The seizure boundary conditions, though derived from instrumented mannequin experiments programmed with clinically recorded convulsion patterns, represent only a subset of the diverse seizure types and severities experienced by pregnant individuals with epilepsy. The computational framework does not incorporate potential complications such as placental abruption, umbilical cord compression, or alterations in amniotic fluid volume that could modify protective capacity in clinical scenarios.

The assumption of uniform cerebrospinal fluid properties throughout the ventricular system and subarachnoid space neglects potential spatial variations in composition or flow patterns that might influence force transmission pathways. The injury thresholds used for comparison in this study were derived predominantly from adult neural tissue studies, and the appropriateness of applying these values to fetal tissue is questionable. Fetal neural tissue exhibits distinct mechanical and biological properties including higher water content, incomplete myelination, different cellular density, and ongoing developmental processes that may alter injury susceptibility in ways not captured by adult-derived thresholds. The absence of validated fetal-specific injury criteria represents a fundamental limitation when attempting to assess whether predicted stress values are truly within safe ranges. The finite element mesh resolution, while sufficient for capturing global biomechanical responses, may not resolve fine-scale stress concentrations at material interfaces or in regions of complex geometry. The contact formulation between smoothed particle hydrodynamics fluid particles and finite element structural surfaces employs penalty-based methods that introduce numerical parameters requiring calibration, and alternative contact algorithms might yield quantitatively different force distributions. The simulation duration captured only the acute mechanical response during traumatic loading, whereas longer-term biological responses involving inflammatory cascades or developmental alterations remain outside the scope of purely mechanical analysis.

It is important to acknowledge that maternal epilepsy is not purely a mechanical event. Seizures involve complex neurobiological and inflammatory processes, including elevated maternal cortisol levels, transient hypoxia, alterations in placental blood flow, and release of inflammatory cytokines, all of which may independently contribute to fetal brain stress and developmental outcomes. The present computational model exclusively captures the mechanical component of maternal seizures; specifically, the convulsive body movements transmitted through the uterus and amniotic fluid to the fetus. Biological factors such as inflammation, neurochemical changes, and metabolic disturbances are not represented in this framework. Consequently, the stress predictions reported here reflect only the mechanical loading component and should not be interpreted as a complete assessment of fetal risk during maternal seizure events. Future research could benefit from integrating mechanical modeling with physiological parameters measured from animal models to provide a more comprehensive understanding of the multifactorial nature of fetal brain injury during maternal epilepsy.

Despite these limitations, the computational framework provides useful insights into force transmission mechanisms through nested protective layers and establishes quantitative baselines for fetal vulnerability assessment during maternal trauma.

### 4.4. Future Directions for Investigation

Several promising directions for future research emerge from this investigation. Extension of the computational framework to multiple gestational ages would enable characterization of how protective efficacy evolves throughout pregnancy as amniotic fluid volume changes, fetal size increases, and tissue material properties mature. Incorporation of patient-specific anatomical geometries derived from magnetic resonance imaging would permit investigation of how variations in fetal positioning, uterine shape, and placental location influence force transmission pathways and stress distributions. Integration of more sophisticated constitutive models capturing the anisotropic and heterogeneous material properties of biological tissues would improve prediction accuracy, particularly for complex structures such as the placenta and umbilical cord. Coupling of mechanical analysis with biological models of cellular damage and inflammatory response would enable prediction of longer-term consequences beyond acute mechanical insults.

To contextualize the boundary conditions employed in this investigation, comparison of the recorded kinematic parameters against those reported in related computational studies of fetal biomechanics was attempted. However, the computational prenatal biomechanics represents a largely neglected area of research. Based on available literature, no prior computational studies have reported kinematic boundary conditions specifically derived from maternal seizure events, precluding direct parameter validation against established reference values. This absence of comparable datasets highlights a significant gap in the prenatal biomechanics literature and underscores the exploratory nature of the present work. Future research establishing standardized kinematic databases for various maternal trauma scenarios, including seizure events, would substantially benefit the field and enable more rigorous model validation.

Development of reduced-order models or surrogate modeling approaches could enable rapid assessment of fetal vulnerability across diverse trauma scenarios without requiring full computational simulations for each case. Such tools would have practical clinical utility for risk stratification and decision support in emergency settings involving pregnant trauma patients. Experimental validation studies using instrumented physical models or animal models would provide essential data for verifying computational predictions and refining model parameters. Investigation of interventional strategies such as positioning recommendations or external support devices could leverage the computational framework to optimize protective measures for high-risk pregnancies. Finally, extension of the methodology to other prenatal trauma scenarios including motor vehicle accidents and falls would broaden the applicability of the nested fluid–structure interaction framework and contribute to comprehensive understanding of fetal mechanical protection across the spectrum of maternal traumatic events encountered in clinical practice.

## 5. Conclusions

The central contribution of this investigation is the demonstration of hierarchical force attenuation through the nested fluid protection architecture, rather than the determination of absolute stress magnitudes. Computational modeling suggests that mechanical loads applied to the maternal body are progressively diminished as they propagate through successive protective layers: from the external environment through the uterine wall, across the amniotic fluid, through the fetal body, across the cerebrospinal fluid space, and into brain tissue. This sequential damping pattern is evidenced by the stark contrast between separation dynamics at different hierarchical levels. The model predicted that uterus-to-fetus distances undergo large-amplitude oscillations ranging from approximately twenty to one hundred eighty percent of baseline separation, whereas brain-to-skull distances remained tightly constrained, varying by less than six percent throughout identical loading scenarios. This quantitative disparity demonstrates that the majority of mechanical energy is dissipated before reaching the fetal cranial system, and this comparative finding is more robust than absolute stress predictions because it is less sensitive to uncertainties in material property assignments and injury threshold applicability.

The findings of this investigation should be interpreted as preliminary computational predictions requiring experimental and clinical validation. The model has not been benchmarked against physical measurements, the material properties for fetal tissues involve substantial uncertainty, and the injury thresholds used for contextual comparison were derived from adult neural tissue studies whose applicability to developing fetal tissue remains unestablished. The primary value of this work lies in establishing a computational framework for systematic analysis of force transmission through nested protective layers and in demonstrating the pattern of hierarchical attenuation, rather than in providing clinically actionable safety assessments. Future experimental studies using phantom models, animal systems, or validated biomechanical measurements are necessary to confirm whether the relative force reductions predicted computationally translate to meaningful protection against fetal neural injury in clinical scenarios.

## Figures and Tables

**Table 1:** Summary of boundary condition parameters derived from instrumented mannequin seizure simulation (*n* = 75 signals).

Parameter	Value (Mean ± Std)	Notes
Peak angular velocity (roll)	9.5±1.1 rad/s	Range: [−8, 10] rad/s
Peak angular acceleration (roll)	5.0±2.0 rad/s^2^	Derived from roll velocity
Angular displacement range	0.50±0.10 rad	Peak-to-peak roll excursion
Dominant frequency	0.5±0.1 Hz	Estimated from oscillations
Simulation duration (T_0_)	60.0 s	Total recording time

**Table 2:** Summary of material properties, literature sources, and key assumptions for model components.

Component	Property	Value/Range	Source/Assumption
Amniotic Fluid	Density	≈1000 kg/m^3^	Water approximation
	Dynamic viscosity	≈0.001 Pa·s	Water approximation
Cerebrospinal Fluid	Density	≈1000 kg/m^3^	Water approximation
	Dynamic viscosity	≈0.001 Pa·s	Water approximation
Fetal Brain	Shear modulus	Reduced vs. adult	Extrapolated from adult data; accounts for incomplete myelination
	Viscoelastic parameters	Estimated	Limited fetal-specific data available
Fetal Skull	Elastic modulus	Reduced vs. adult	Reflects incomplete ossification
Uterine Wall	Viscoelastic properties	From literature	Human physiological measurements

## Data Availability

The original contributions presented in this study are included in the article. Further inquiries can be directed to the corresponding author.

## Abbreviations

The following abbreviations are used in this manuscript:

FSI	Fluid-Structure Interaction
FEM	Finite Element Method
SPH	Smoothed Particle Hydrodynamics
